# Prevalence of antiretroviral therapy treatment failure among HIV-infected pregnant women at first antenatal care: PMTCT Option B+ in Malawi

**DOI:** 10.1371/journal.pone.0209052

**Published:** 2018-12-13

**Authors:** Maganizo B. Chagomerana, William C. Miller, Jennifer H. Tang, Irving F. Hoffman, Bryna J. Harrington, Bethany DiPrete, Shaphil Wallie, Allan Jumbe, Laura Limarzi, Mina C. Hosseinipour

**Affiliations:** 1 UNC Project-Malawi, Kamuzu Central Hospital, Lilongwe, Malawi; 2 Division of Epidemiology, College of Public Health, The Ohio State University, Columbus, Ohio, United States of America; 3 Department of Obstetrics and Gynecology, The University of North Carolina at Chapel Hill, Chapel Hill, North Carolina, United States of America; 4 Department of Medicine, The University of North Carolina at Chapel Hill, Chapel Hill, North Carolina, United States of America; 5 Department of Epidemiology, Gillings School of Global Public Health, The University of North Carolina at Chapel Hill, Chapel Hill, North Carolina, United States of America; Vanderbilt University Medical Center, UNITED STATES

## Abstract

**Background:**

In Malawi’s PMTCT Option B+ program, HIV-infected pregnant women who are already receiving ART are continued on their current therapy regimen without testing for treatment failure at the first antenatal care (ANC) visit. HIV RNA screening at ANC may identify women with treatment failure and ensure that viral suppression is maintained throughout the pregnancy.

**Methods:**

We conducted a cross-sectional study of HIV-infected pregnant women who had been receiving ART for at least 6 months at the first ANC visit under the PMTCT Option B+ program at Bwaila Hospital in Lilongwe, Malawi from June 2015 to December 2017. Poisson regression models with robust variance were used to investigate the predictors of ART treatment failure defined as viral load ≥1000 copies/ml.

**Results:**

The median age of 864 women tested for ART failure was 31.1 years (interquartile range: 26.9–34.5). The prevalence of treatment failure was 7.6% (95% confidence interval (CI): 6.0–9.6). CD4 cell count (adjusted prevalence ratio (aPR) = 0.57; 95% CI: 0.50–0.65) was strongly associated with treatment failure.

**Conclusion:**

The low prevalence of treatment failure among women presenting for their first ANC in urban Malawi demonstrates success of Option B+ in maintaining viral suppression and suggests progress towards the last 90% of the UNAIDS 90-90-90 targets. Women failing on ART should be identified early for adherence counseling and may require switching to an alternative ART regimen.

## Introduction

Antiretroviral therapy (ART) failure is an emerging challenge in the fight against the HIV/AIDS pandemic. HIV-infected individuals may develop ART failure because of primary infection with drug-resistant HIV strain or poor adherence to therapy. Often characterized by persistently high levels of viral load (VL), ART failure can lead to the development of HIV drug resistant (HIVDR) strain.[1] When HIV-infected people with drug resistance are not promptly detected and switched to an alternative and effective ART regimen, they are likely to transmit the resistant strain to their sexual partners and infants. When HIV-infected pregnant women transmit drug resistant HIV to their infants,[2, 3] both the mother and child have impaired ART efficacy.[4]

The prevention of mother-to-child transmission (PMTCT) Option B+ program in Malawi increased the uptake of ART among pregnant women by 748% after one year of implementation [5]. The dramatic increase in ART uptake among pregnant and breastfeeding women during the Option B+ era has resulted in a 67% decrease in mother-to-child transmission.[6] However, the program continues to experience poor retention in care, especially during the postpartum period.[7–9] Most women who remain in care have unmonitored VL because HIV RNA testing is not performed on their scheduled dates, which according to Malawi’s Ministry of Health (MOH) guidelines should be 6 and 24 months after ART initiation, and every 2 years thereafter.[10] As a result, women who are in care but have developed treatment failure may go unnoticed.

Detection of treatment failure early in pregnancy among women who are already on therapy is essential for achieving maximal PMTCT. In Malawi’s Option B+ program, HIV-infected pregnant women who are already receiving ART are continued on therapy without testing for treatment failure at the first antenatal care (ANC) visit, an opportune time for early detection of treatment failure in pregnancy. In this study, we estimated the prevalence of treatment failure among women who were already receiving ART when presenting for their first ANC visit at a large urban hospital in Lilongwe, Malawi. We also identified demographic and HIV testing and treatment factors associated with treatment failure.

## Methods

### Study design, population and setting

We conducted a cross-sectional study of HIV-infected pregnant women who were receiving ART at the first ANC visit under the PMTCT Option B+ program at Bwaila Hospital in Lilongwe, Malawi from June 2015 to December 2017. All HIV-infected pregnant women who had been receiving ART for ≥ 6 months at the first ANC visit and were aged 16 years or older (adults and emancipated minors) were eligible for treatment failure screening. We excluded all pregnant women who did not provide written informed consent to participate in the study.

This study was approved by the National Health Sciences Research Committee of Malawi and the Institutional Review Board at the University of North Carolina at Chapel Hill. All participants were required to sign a written informed consent before study participation.

### Study procedures

All women who consented to be enrolled in the study were interviewed in person at the first ANC visit to collect HIV testing history and demographic and treatment information. HIV status and ART exposure documented during the interviews were cross-checked with documentation in the participant’s health passport (a government-issued document that contains information on general medical history, diagnoses, treatments, antenatal consultations and deliveries) and the participant’s ART mastercard (a tool for recording demographic and ART treatment information for persons receiving ART).

In Malawi, the guidelines for providing HIV services has evolved over the years. The most notable change during our study period was the recommendation to start ART as soon as possible regardless of WHO clinical stage or CD4 cell count in May 2016 –the test-and-treat approach.[11] However, the management of people with ART failure did not change. HIV-infected persons who are identified as having developed treatment failure are not immediately started on 2^nd^ line therapy. Under routine care, people identified as experiencing treatment failure during any of the scheduled VL testing times receive intensive adherence counselling before a repeat VL testing after 3 months.[10, 11] Individuals with VL ≥1000 copies /ml during the repeat testing are started on 2^nd^ line therapy while those with VL <1000 copies/ml are continued on intensive adherence counselling.

HIV RNA testing was conducted at the UNC Project-Malawi clinical research laboratory using the Abbott M2000 system with a lower limit of quantification of 40 copies/ml for samples of 0.6 ml. Samples with detectable VL that was <40 copies/ml were indicated as “<40 copies/ml”. Samples with no detectable VL were designated as “undetectable”.

### Variable definitions and classification

The primary outcome was treatment failure at first ANC visit, defined as VL ≥1000 copies/ml to align with the Malawi Ministry of Health guidelines. Variables considered for assessment as risk factors for treatment failure were selected based on clinical relevance and literature review. The candidate predictors identified for assessment were age, pregnancy trimester at first ANC visit, level of education, marital status, parity, WHO clinical stage, partner’s HIV status, CD4 cell count at enrollment, reason for starting ART, current ART regimen, duration receiving ART, and ART adherence. Some women started ART during a previous pregnancy or during breastfeeding as part of PMTCT program, while others started prior to the current pregnancy due to health conditions that met national guidelines for ART initiation.[10] ART adherence assessment was based on recorded pill counts in health passport or ART mastercard for the past 3 months prior to the first antenatal visit. In Malawi, a person is declared to have good adherence to ART if they have taken ≥95% of doses in the prescribed interval.[10]

We restricted categorization of all candidate predictors (except partner’s HIV status and ART regimen being taken) to two categories to minimize sparse data. Briefly, we categorized the variables as follows: age (<30 years vs ≥30 years), pregnancy trimester (1^st^ or 2^nd^ trimester vs 3^rd^ trimester), level of education (no school/primary vs any secondary/tertiary), marital status (currently married vs not currently married), parity 0–1 child vs ≥2 children), WHO clinical stage (Stage 1 vs Stage 2, 3 and 4), partner’s HIV status (negative, positive, and unknown), CD4 cell count at enrollment (<500 cells/mm^3^ vs ≥500 cells/mm^3^), reason for starting ART (previous pregnancy vs ART for health), ART regimen being taken (tenofovir, lamivudine and efavirenz (TDF/3TC/EFV), PI-based 2^nd^ line regimens, and other non-nucleoside reverse-transcriptase inhibitors (NNRTI)-based regimens), duration receiving ART (6–12 months vs ≥12 months), and ART adherence (pill count suggestive of <95% adherence vs ≥95%). In all regression models, CD4 cell count was used as a rescaled continuous variable.

### Statistical analyses

The primary objective of this analysis was to estimate the prevalence of treatment failure. We calculated the proportion of women with treatment failure and the corresponding two-sided 95% confidence interval (CI). We used proportions and medians to summarize the distribution of categorical variables and continuous variables, respectively. Fisher’s exact tests were used to assess the association between predictors and prevalence of treatment failure.

High viral loads among HIV-infected persons who are receiving ART with adequate adherence can be due to primary infection with a drug-resistant strain or poor adherence that led to development of drug resistance. CD4 counts indicate poorer immune function, and starting ART at low CD4 counts may increase the risk of developing drug resistant HIV.[12] Those with low CD4 at a more advanced stage of HIV disease, typically have higher HIV RNA and hence suppression may be more difficult to achieve. The longer suppression period in the setting of drug exposure may allow the evolution of drug resistant virus. To assess if there were some pregnant women who may have transmitted resistant strain, we stratified our analysis of the association between duration receiving ART and treatment failure by CD4 cell count (<500 cells/mm^3^ or ≥500 cells/mm^3^). We also assessed if there was interaction between ART duration and CD4 cell count.

Poisson regression models with robust variance were used to estimate unadjusted and adjusted prevalence ratios (uPR and aPR) and 95% CI of the association between each predictor and prevalence of treatment failure. We used Poisson regression with robust variance estimates due to problems with consistent convergence of log-binomial regression models.[13, 14] In the multivariable model, we included only those predictors that yielded a p-value <0.50 in univariable analyses. Our inclusion criterion for variables in the multivariable model was less stringent (higher p-value threshold) to ensure that all potentially important variables were retained given the exploratory nature of the analysis.[15]

## Results

From June 2015 to December 2017, 1566 women were screened for enrolment and 639 women (40.8%) were not eligible. Of the 927 eligible pregnant women, 63 women (6.8%) refused consent to participate in the study. The remaining 864 pregnant women who had been receiving ART for at least 6 months were tested for treatment failure at their first ANC visit.

Among the women who were tested for treatment failure, the median age at first ANC visit was 31.1 years (interquartile range (IQR): 26.9–34.5) and median gestation age was 22 weeks (IQR: 18–27) (Table 1). Eighty-three percent (n = 718) were in the 1^st^ or 2^nd^ trimester of pregnancy, 803 (92.9%) were married, 699 (80.9%) were in WHO Clinical Stage 1, 519 (61.3%) started ART for health, and 829 (98.9%) had pill count suggestive of ≥95% adherence for the past 3 months. The median duration of ART was 3.9 years (IQR: 2.2–5.8) and most women (n = 762, 90.6%) were receiving ART for ≥ 1 year. Only 240 women (27.9%) reported ever having an HIV viral load test.

**Table 1:** Participants’ characteristics at the first antenatal care.

Characteristic		Total (N = 864) n (%)
Age (years)		
	16–29	341 (39.5)
	≥30	523 (60.5)
Gestational age		
	1^st^ or 2^nd^ trimester	718 (83.4)
	3^rd^ trimester	143 (16.6)
Education		
	No school or Primary	539 (62.5)
	Secondary or Tertiary	324 (37.5)
Marital status		
	Currently married	803 (92.9)
	Not currently married	61 (7.1)
Parity		
	0–1 child	174 (20.2)
	≥2 children	688 (79.8)
WHO clinical stage		
	Stage 1	699 (80.9)
	Stage 2, 3 and 4	165 (19.1)
Partner HIV status		
	Negative	181 (21.1)
	Positive	477 (55.6)
	Unknown	200 (23.3)
Ever had HIVRNA test		
	No	619 (72.1)
	Yes	240 (27.9)
Reason for starting ART		
	Previous pregnancy / Breastfeeding	327 (38.6)
	ART for health	519 (61.4)
ART regimen		
	TDF/3TC/EFV	791 (93.7)
	2^nd^ line PI-based	22 (2.6)
	Other NNRTI-based	31 (3.7)
Pill count suggestive of 95% adherence		
	No	9 (1.1)
	Yes	829 (98.9)
ART duration		
	≥12 months	762 (90.6)
	6–12 months	79 (9.4)
CD4 cell count		
	≥500 cells/mm^3^	500 (58.5)
	<500 cells/mm^3^	355 (41.5)
		Median (IQR)
	ART duration (years)	3.9 (2.2–5.8)
	CD4 cell count (/100 cells)	5.41 (3.91–6.98)

Observations with missing values: gestational age = 3, education = 1, parity = 2, CD4 cell count = 9, ever had HIVRNA test = 5, partner HIV status = 6, reasons for starting ART = 18, ART Regimen = 20, ART duration = 23, and pill count suggestive of 95% adherence = 26.

Of the 864 women, 66 had VL ≥1000 copies/mm^3^, resulting in overall prevalence of ART treatment failure of 7.6% (95% CI: 6.0–9.6). The median VL for women with VL ≥1000 copies/ml was 10,434 copies/ml (IQR: 2,405–44,511). Among the 768 women with VL <1000 copies/ml, 82.7% (n = 660) had an undetectable VL, 11.9% (n = 95) had a VL<40 copies/ml, and 5.4% (n = 43) had a VL between 40 and 1000 copies/ml. The median VL for women with VL between 40 and 1000 copies/ml was 254 copies/ml (IQR: 72–450).

The prevalence of treatment failure was higher among women who had a CD4 cell count <500 cells/mm^3^ (14.9%) compared to women who had a CD4 cell count ≥500 cells/mm^3^ (2.6%). The prevalence of treatment failure was also higher among women who had been receiving ART for <1 year (13.9%) than those who had been receiving ART ≥1 year (7.2%). In unadjusted analyses, a 100-cell increase in CD4 cell count was associated with a decrease in prevalence of treatment failure at the time of first ANC visit (uPR = 0.55; 95% CI: 0.49–0.63). The prevalence of treatment failure among women who had been receiving ART for 6–12 months was 1.93 (95% CI: 1.05–3.53) times as high as that of women who had been receiving ART for at least 1 year. However, the prevalence of treatment failure did not differ by age, gestational age, education, marital status, parity, WHO clinical stage, partner HIV status, ever having an HIVRNA test, reason for starting ART, and ART regimen (Table 2). We did not calculate PRs for ART adherence because none of the women who had pill counts suggestive of <95% adherence had developed treatment failure.

**Table 2:** Association between participants’ characteristics and prevalence of treatment failure at first antenatal care visit.

		Viral load			
Characteristic		≥1000 copies/ml N = 66 n (%)	<1000 copies/ml N = 798 n (%)	Unadjusted Prevalence Ratio	Adjusted Prevalence Ratio
Age (years)					
	16–29	26 (7.6)	315 (92.4)	1.0	–
	≥30	40 (7.7)	483 (92.3)	1.00 (0.62–1.61)	
Gestational age					
	1^st^ or 2^nd^ trimester	56 (7.8)	662 (92.2)	1.0	–
	3^rd^ trimester	10 (7.0)	133 (93.0)	0.90 (0.47–1.72)	
Education					
	No school or Primary	45 (8.4)	494 (91.6)	1.0	1.0
	Secondary or Tertiary	21(6.5)	303 (93.5)	0.78 (0.47–1.28)	0.71 (0.45–1.13)
Marital status					
	Currently married	59 (7.4)	744 (92.6)	1.0	1.0
	Not currently married	7 (11.5)	54 (88.5)	1.56 (0.75–3.27)	1.39 (0.74–2.59)
Parity					
	0–1 child	13 (7.5)	161 (92.5)	1.0	–
	≥2 children	53 (7.7)	635 (92.3)	1.03 (0.58–1.85)	
WHO clinical stage					
	Stage 1	52 (7.4)	647 (92.6)	1.0	–
	Stage 2, 3 and 4	14 (8.5)	151 (91.5)	1.14 (0.64–2.01)	
Partner HIV status					
	Negative	15 (8.3)	166 (91.7)	1.0	
	Positive	34 (7.1)	443 (92.9)	0.86 (0.48–1.54)	–
	Unknown	17 (8.5)	183 (91.5	1.03 (0.53–1.99)	
Ever had HIVRNA test					
	No	44 (7.1)	575 (92.9)	1.0	1.0
	Yes	22 (9.2)	218 (90.8)	1.29 (0.79–2.10)	0.99 (0.61–1.59)
Reason for starting ART					
	Previous pregnancy / Breastfeeding	20 (6.1)	307 (93.9)	1.0	1.0
	ART for health	45 (8.7)	474 (91.3)	1.42 (0.85–2.36)	0.85 (0.54–1.34)
ART regimen					
	TDF/3TC/EFV	59 (7.5)	732 (92.5)	1.0	1.0
	2^nd^ line PI-based	3 (13.6)	19 (56.4)	1.83 (0.62–5.39)	1.07 (0.43–2.67)
	Other NNRTI-based	4 (12.9)	27 (87.1)	1.73 (0.67–4.46)	1.53 (0.64–3.64)
Pill count suggestive of 95% adherence					
	No	0 (0.0)	9 (100.0)	–	–
	Yes	62 (7.5)	767 (92.5)		
ART duration					
	≥12 months	55 (7.2)	707 (92.8)	1.00	1.0
	6–12 months	11 (13.9)	68 (86.1)	1.93 (1.05–3.53)	1.23 (0.69–2.20)
CD4 cell count					
	≥500 cells/mm^3^	13 (2.6)	487 (97.4)	–	–
	<500 cells/mm^3^	53 (14.9)	302 (85.1)		
		Median (IQR)	Median (IQR		
ART duration (years)		3.8 (1.4–6.2)	3.9 (2.2–5.8)	–	–
CD4 cell count (/100 cells)		2.56 (1.33–4.36)	5.59 (4.19–7.09)	0.55 (0.49–0.63)	0.57 (0.50–0.65)

Observations with missing values: gestational age = 3, education = 1, parity = 2, CD4 cell count = 9, ever had HIVRNA test = 5, partner HIV status = 6, reasons for starting ART = 18, ART Regimen = 20, ART duration = 23, and pill count suggestive of 95% adherence = 26.

When stratified by CD4 cell count, prevalence of treatment failure was similar between women who had been receiving ART for ≥ 1 year (2.7%) and those who had been receiving ART for <1 year (2.5%) among those with a CD4 cell count ≥500 cells/mm^3^. For women with a CD4 cell count <500 cells/mm, the prevalence of treatment failure was lower among women who had been receiving ART for ≥ 1 year (14%) compared to women who had been receiving ART for <1 year (27%). There was no evidence of interaction between ART duration and CD4 cell count.

Our multivariable model included education, marital status, CD4 cell count, ever having an HIVRNA test, reason for starting ART, ART regimen, and ART duration. Adjusting for all other variables in the model, only CD4 cell count was significantly associated with treatment failure at the time of first antenatal visit, aPR = 0.57; 95% CI: 0.50–0.65 (Table 2).

## Discussion

In this cross-sectional study, few women receiving ART for at least 6 months had developed treatment failure at the time of their first ANC visit. The presence of women with treatment failure at first ANC visit is a concern in our setting because in routine antenatal care, pregnant women already receiving ART are continued on therapy without VL testing. To be tested for VL during pregnancy, pregnant women must also be attending routine outpatient ART clinic and have reached a designated VL test time-point as per Malawi’s MOH guidelines for HIV management (6 and 24 months after ART initiation and every 2 years thereafter).[10] HIV-infected pregnant women with high viremia are at risk of transmitting HIV to their infants, including potentially a drug resistant strain.[4]

Comparing findings from studies of viral suppression is generally difficult because of differences in thresholds of viral suppression used and duration of therapy before evaluation. Nevertheless, most studies have demonstrated good viral suppression among women who initiated ART through Option B+. Viral suppression (< 400 copies/ml) of 81% was reported from a cohort study of women initiated on ART during Option B+ program and followed over 4 years in Uganda, with almost 90% viral suppression among women who were retained in care.[16] After six months of ART initiation under Option B+ program in another cohort of pregnant and breastfeeding women in Malawi, 84% achieved VL <1000 copies/ml.[17] In a cohort in South Africa, 91% of pregnant women achieved viral suppression (<1000 copies/ml) by delivery.[18]

The low percentage of women with evidence of treatment failure at the first ANC visit highlights the progress that Malawi has made towards achieving the last 90% of the UNAIDS 90-90-90 targets among women for control of the HIV epidemic.[19] Among our participants, less than 10% of women had a VL ≥1000 copies/ml, meaning that most pregnant women who had been receiving ART for ≥6 months presenting to ANC were virally suppressed. Our finding on the proportion of viral suppression is similar to the results from the Malawi Population-Based and HIV Impact Assessment survey.[20] Overall, 92% of women who were receiving ART during the survey were virally suppressed (VL<1000 copies/ml), although the proportions varied across age categories.

A higher proportion of women with low CD4 counts (<500/mm^3^) had ART treatment failure at first antenatal visit compared to women with higher CD4 counts. The association between low CD4 cell count and ART failure is not surprising, and including CD4 count improves models of viral failure.[21] As CD4 cell count decreases, HIV viral replication increases leading to high levels of VL.[22] The persistence of VL ≥1000 copies/ml after receiving ART for at least 6 months merits further investigation. Possibilities for persistent high VL include failure to suppress secondary to extremely high viral load, poor adherence with or without acquired drug resistance, or transmitted drug resistance. Our cohort had all been on therapy for at least 6 months and most women who initiate ART achieve viral suppression within 3 months, making the first scenario less likely.[23]

Inadequate adherence to ART is a primary cause of failure to reach viral suppression in the first year. Therefore, the higher prevalence of treatment failure among women with a CD4 cell count <500 cells/mm^3^ who had been receiving ART for <1 year compared to women with similar CD4 cell count but had been receiving ART for at least 1 year may suggest the existence of transmitted non-nucleoside reverse-transcriptase inhibitors (NNRTI) resistance. Transmitted NNRTI resistance has been documented in the sub-Saharan region[24–27] and may compromise the response to first-line therapy in our population. In addition, viremic episodes in the postpartum period may occur frequently after viral suppression,[28, 29] so it is important to encourage consistent ART adherence and follow-up with clinicians to prevent development of resistance that can lead to horizontal or vertical HIV transmission.

Age, gestational age, education, marital status, parity, WHO clinical stage, partner HIV status, reason for starting ART, and ART regimen are known to be associated with treatment failure.[30–32] However, none of these factors were associated with the prevalence of treatment failure in our population of pregnant women. The failure to identify these characteristics as risk factors in our study may be due to homogeneity of our participant population. For example, among the women tested for treatment failure, over 98% had pill counts suggestive of 95% adherence, 94% were on the first-line ART regimen, and 81% were in WHO Stage 1. Low CD4 count was associated with treatment failure in our population and could be used as part of an algorithm that would identify pregnant women who are at high risk of treatment failure from this population. Malawi’s HIV treatment guidelines are moving away from routine CD4 measurements, similar to many countries using a test-and-treat approach, which renders CD4 counts less useful predictors of treatment failure.[10, 33] More recently, the role of CD4 testing for identification of advanced disease at initiation[34, 35] and use of differentiated pathways for ART monitoring[36] through customized packages of services for patient groups may revive interest in CD4 testing for failure algorithm testing.

In this study, over 98% of pregnant women had pill counts suggestive of 95% adherence at the first ANC visit. Considering the reported sub-optimal retention in care rates among HIV-positive mothers in the Malawi’s PMTCT program,[7, 8] this high rate of adherence is surprising. The observed high adherence rate may have resulted from over reporting on pill count during clinic visits. In this study, pill counts were based on records in ART mastercards or health passports prior to the first ANC visit and therefore could not be verified. The high adherence rate may be secondary to selection bias as we enrolled women who were attending an ANC visit and therefore were more likely to be accessing ART and adherent than women who did not attend an ANC visit. In addition, since our study was performed in an urban setting, our study population may have had better access to ART than women living in rural settings. In Malawi, about 31% of mothers who start ART become lost to follow-up (LTFU) within 12 months of treatment initiation.[37] Pregnant and breastfeeding women who are LTFU in the continuum of PMTCT care are likely to have stopped taking ART or be less adherent to treatment if still taking medication. However, many women (61%) in this study started ART for health. In Malawi, women who start ART due to poor health are less likely to be LTFU and more likely to be adherent to treatment than women who start ART because of pregnancy, which may also account for our high adherence rate.[8]

Although all HIV-infected adults who initiate ART are supposed to have HIV RNA testing in routine care at 6 and 24 months, and every 2 years thereafter in Malawi,[10] close to 40% miss these routine tests and therefore have unmonitored VL.[38] In our population of pregnant women, less than one-third of women reported ever having an HIV RNA test. Recent MOH HIV quarterly report highlights program wide challenges with VL testing including poor tracking of samples and results and delays in sample processing.[39] When pregnant, women who have unmonitored VL are at high risk of transmitting HIV to their infants if not identified early in pregnancy. Moreover, among pregnant women receiving ART, VL monitoring based on gestational age may be more informative to predict viremia at delivery than the typical guidelines which use time since ART initiation.[40] In settings with poor roll-out of VL monitoring, VL testing at first antenatal visit provides an opportunity to identify women who have developed treatment failure and intervene through adherence counseling or switching to an alternative and effective regimen.

Our results demonstrate that some pregnant women receiving ART were experiencing treatment failure at the time of their first ANC visit. With universal ART, the number of pregnant women reporting for first ANC already receiving ART is likely to increase. In the absence of wide access to VL testing through routine care, targeted antenatal HIV RNA testing provides an opportunity to identify and intervene in cases of treatment failure to to minimize the risk of mother-to-child transmission.

## Supporting information

S1 File (CSV)
